# Prediction models for cognitive impairment in middle-aged patients with cerebral small vessel disease

**DOI:** 10.3389/fneur.2025.1462636

**Published:** 2025-02-11

**Authors:** Wei Zheng, Xiaoyan Qin, Ronghua Mu, Peng Yang, Bingqin Huang, Zhixuan Song, Xiqi Zhu

**Affiliations:** ^1^Department of Radiology, Nanxishan Hospital of Guangxi Zhuang Autonomous Region, Guilin, China; ^2^Graduate School, Guilin Medical University, Guilin, China; ^3^Philips (China) Investment Co., Ltd., Guangzhou Branch, Guangzhou, China; ^4^Department of Radiology, Affiliated Hospital of Youjiang Medical University for Nationalities, Baise, China; ^5^Life Science and Clinical Medicine Research Center, Affiliated Hospital of Youjiang Medical University for Nationalities, Baise, China

**Keywords:** cerebral small vessel disease, cognitive impairment, magnetic resonance imaging, prediction model, radiomics

## Abstract

**Purpose:**

This study aims to develop hippocampal texture model for predicting cognitive impairment in middle-aged patients with cerebral small vessel disease (CSVD).

**Methods:**

The dataset included 145 CSVD patients (Age, 52.662 ± 5.151) and 99 control subjects (Age, 52.576±4.885). An Unet-based deep learning neural network model was developed to automate the segmentation of the hippocampus. Features were extracted for each subject, and the least absolute shrinkage and selection operator (LASSO) method was used to select radiomic features. This study also included the extraction of total intracranial volume, gray matter, white matter, cerebrospinal fluid, white matter hypertensit, and hippocampus volume. The performance of the models was assessed using the areas under the receiver operating characteristic curves (AUCs). Additionally, decision curve analysis (DCA) was conducted to justify the clinical relevance of the study, and the DeLong test was utilized to compare the areas under two correlated receiver operating characteristic (ROC) curves.

**Results:**

Nine texture features of the hippocampus were selected to construct radiomics model. The AUC values of the brain volume, radiomics, and combined models in the test set were 0.593, 0.843, and 0.817, respectively. The combination model of imaging markers and hippocampal texture did not yield improved a better diagnosis compared to the individual model (*p* > 0.05).

**Conclusion:**

The hippocampal texture model is a surrogate imaging marker for predicting cognitive impairment in middle-aged CSVD patients.

## 1 Introduction

In recent years, there has been a growing emphasis on the vascular cognitive impairment stemming from cerebral small vessel disease (CSVD). CSVD has traditionally been linked to disorders of the perforator artery and cerebral microcirculation. However, there is now a consensus that CSVD and neurodegeneration coexist and mutually exacerbate disease progression (1). Consequently, brain atrophy is recognized as a characteristic imaging marker of CSVD (1, 2). Notably, the atrophy of the hippocampus has received significant attention due to its vital role in memory, navigation, and cognition (3). Previous studies have indicated that early Alzheimer's disease (AD) is frequently associated with reduced hippocampal subfields displaying various phenotypes (4). Additionally, hippocampal atrophy not only elevates the risk of progression from mild cognitive impairment (MCI) to AD but also contributes to the progressive memory loss in AD patients (5–7). White matter hyperintensity (WMH), brain gray matter, and hippocampal volume have all been established as independent predictors of cognitive impairment in CSVD (8). Furthermore, brain atrophy is often closely associated with the severity or burden of CSVD (9). However, WMH and brain atrophy tend to be relatively mild in middle-aged patients with CSVD. A long-term observational cohort study on middle-aged adults revealed that a higher WMH burden was observed in only 3% of the participants (10). Mu et al. reported that the incidence of WMH was only 2% in individuals aged 51–55 with Fazekas 3, while the incidence increased to 24% in those aged 60–70 (11). Moreover, the incidence of temporal lobe and hippocampal atrophy was 36.3% in individuals aged 51–55, compared to 80% in those aged 60–70 (11). Consequently, further validation is needed to confirm the predictive value of brain atrophy and WMH for cognitive impairment in middle-aged CSVD patients.

With the rapid advancement of image analysis techniques, radiomics has garnered increasing attention in the study of cognitive impairment (12). Radiomics allows for the extraction of high-throughput features closely associated with cell-level heterogeneity indices from segmented regions of the target organ, enabling quantitative analysis of lesion heterogeneity through appropriate models (13). These features can help identify microcirculation and microenvironmental anomalies associated with the disease. Previous studies have demonstrated the effectiveness of hippocampal radiomics in differentiating between Alzheimer's disease (AD) and mild cognitive impairment (MCI) from normal subjects (14–16). We posit that radiomics based on structural MRI of the hippocampus could be leveraged for the prediction of early cognitive impairment in middle-aged patients with CSVD.

This study aims to develop a radiomics model based on three-dimensional (3D) T1-weighted images to predict cognitive impairment in middle-aged patients with CSVD. Additionally, the study constructed and compared three models: the radiomics model, the imaging marker model, and the combined model, to assess the significance of imaging markers of CSVD and hippocampal radiomics in predicting cognitive impairment in middle-aged CSVD patients.

## 2 Materials and methods

### 2.1 Subjects

**Figure 1:**
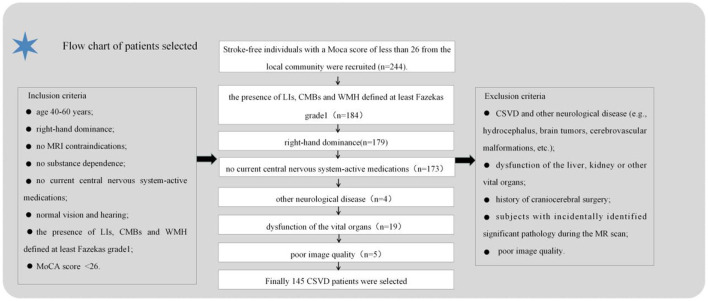
Flowchart of inclusion/exclusion process for subject recruitment.

A community-based cross-sectional study was conducted at Nanxishan Hospital of Guangxi Zhuang Autonomous Region from May 2020 to June 2021, during which 145 CSVD patients with cognitive impairment were recruited from the local community. and CSVD was defined according to previous study (1). Ninety-nine gender-age-and educational matched healthy populations were recruited as control subjects. Demographic information, including gender, age, BMI, and education level, was collected for all participants. The cognitive assessment was performed using the Beijing version of the Montreal Cognitive Assessment (MoCA) by a skilled neuro-radiologist. A MoCA score of <26 indicates cognitive impairment (17). Additionally, they underwent a brain MRI examination within 1 week preceding the evaluations. The process of inclusion and exclusion criteria in the current study is illustrated in Figure 1.

### 2.2 MRI data acquisition

All MRI procedures were conducted using a 3.0T Magnetic Resonance (MR) scanner (Ingenia CX, Philips Healthcare) equipped with a 32-channel head coil. The scan sequences included the following: three-dimensional (3D) T1 fast field echo with repeat time (TR) of 6.4 ms, echo time (TE) of 3.0 ms, field of view (FOV) of 240 × 240 × 180 mm, reconstruction voxel size of 1.1 × 1.1 × 1.1 mm, reconstruction matrix of 400 × 400, and slice thickness of 1.1 mm; 3D T2 spin echo with TR of 2,500 ms, TE of 232 ms, FOV of 250 × 25 × 180 mm, reconstruction voxel size of 1.1 × 1.1 × 1.1 mm, reconstruction matrix of 512 × 512, and slice thickness of 1.1 mm; 3D fluid attenuated inversion recovery with TR of 4,800 ms, TE of 244 ms, FOV of 240 × 240 × 173 mm, reconstruction voxel size of 1.1 × 1.1 × 1.1 mm, reconstruction matrix of 384 × 384, and slice thickness of 1.2 mm. Susceptibility weighted imaging (SWI) with TR of 51 ms, TE of 9.8 ms, FOV of 230 × 189 × 130 mm, reconstruction voxel size of 0.3 × 0.3 × 1 mm, reconstruction matrix of 768 × 768, and slice thickness of 1 mm. Each subject underwent the aforementioned MRI sequences.

### 2.3 Hippocampal segmentation

An Unet-based deep learning neural network model was developed in this study, with the insertion of two modules, semantic-aware normalization (SAN) and semantic-aware whitening (SAW), between the encoder and the decoder to enhance the generalization capability of the model (18). Raw MR scans were input into the hierarchical model, and the probability outputs were obtained using the sigmoid function after the training process. The training data for automated hippocampal segmentation in this study were obtained from a public dataset (https://www.kaggle.com/datasets/sabermalek/mrihs). The detailed procedure of hippocampal segmentation is described in our earlier study (19).

### 2.4 Brain volume features segmentation

**Figure 2:**
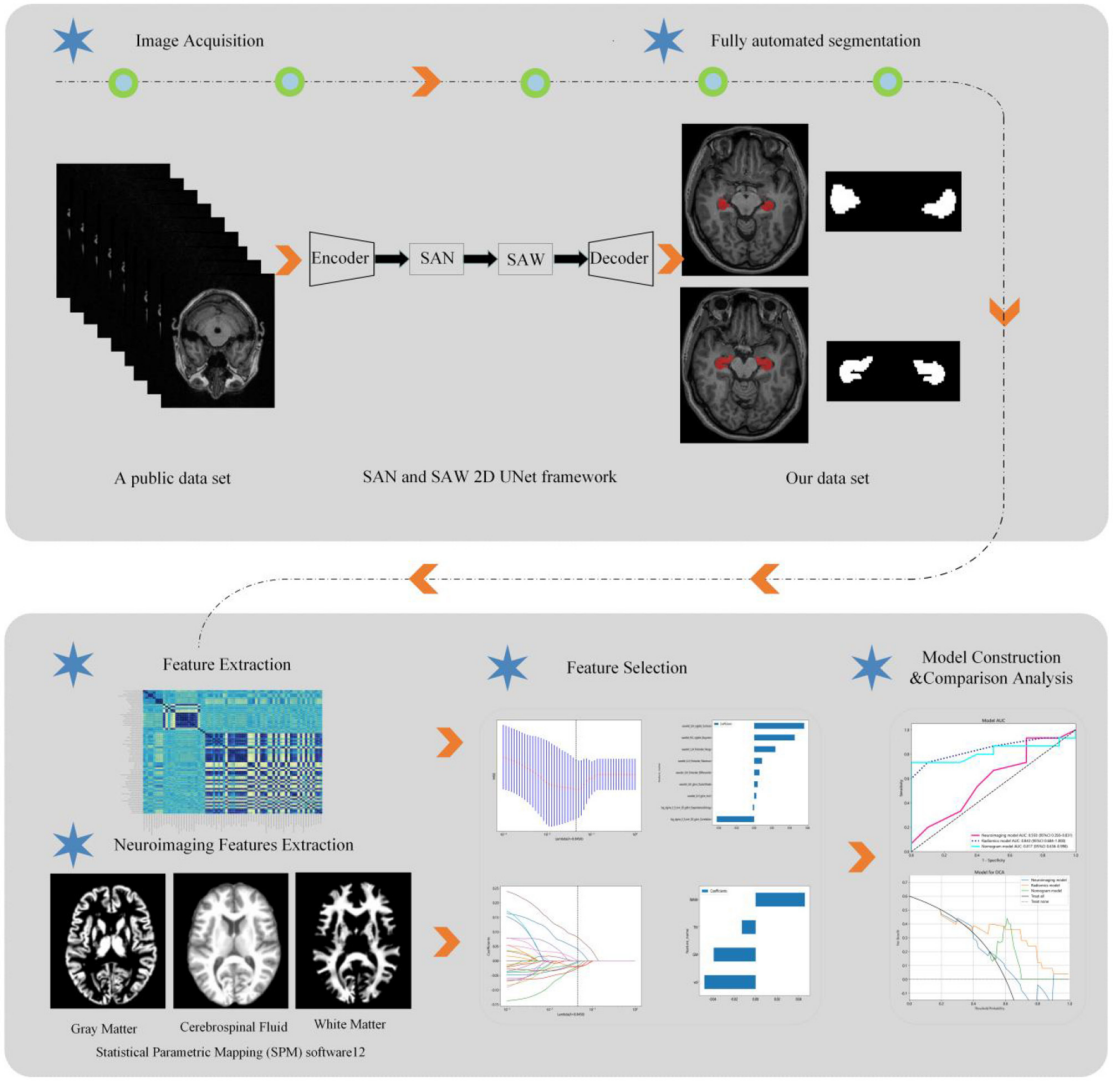
The flowchart shows the process of radiomics and brain volume analysis in CSVD patients.

The CAT12 package was integrated into Statistical Parametric Mapping (SPM) software to post-process our 3D T1-weighted image data. SPM12 was utilized within MATLAB 2018b (MathWorks, Natick, MA). Subsequently, the CAT12 algorithm automatically segmented each structural image into gray matter (GM), white matter (WM), and cerebrospinal fluid. DARTEL normalization was then employed to transform the images into Montreal Neurological Institute (MNI) space. Whole-brain gray matter (GM) volume for statistical analyses was derived by smoothing the normalized GM images with a full width at half maximum of 8 mm. The total intracranial volume (TIV) was computed as the sum of the GM, WM, and cerebrospinal fluid (CSF) volumes. Importantly, group comparisons were solely conducted within a GM mask created by thresholding the mean GM map, including all participants at 0.25 through our automated procedure. Hippocampal volumes (H-VOL) were evaluated using an automated procedure within VolBrain, an online brain segmentation tool (https://volbrain.net). Figure 2 illustrates the radiomics analysis workflow in this study.

### 2.5 Radiomic features extraction and features selection

In this study, radiomic features were extracted using pyradiomics (http://pyradiomics.readthedocs.io) from 3D MRI T1-weighted images. The extracted features included first-order features, gray-level co-occurrence matrix (GLCM), gray level dependence matrix (GLDM), gray-level run length matrix (GLRLM), gray-level size zone matrix (GLSZM), neighboring gray tone difference matrix (NGTDM), and shape features. First-order features describe the distribution of pixel intensities in an image, encompassing mean, variance, and skewness. GLCM features capture spatial relationships and intensity distribution among pixels, while GLDM features represent pixel intensity distribution. GLRLM features characterize the periodicity and directionality of texture, and GLSZM features depict spatial relationships and intensity distribution between adjacent pixels. NGTDM features represent an improved form of gray-level co-occurrence matrix features, and shape features illustrate the contours and shape information of objects in an image. Each feature value was normalized using *Z*-scores [(*x* – μ)/σ], where x refers to the value of the feature, μ represents the average value of the feature for all patients in the cohort, and σ indicates the corresponding standard deviation, thus removing the unit limits of each feature before applying the machine learning model for classification. Additionally, all subjects were randomly divided into training and test sets in a ratio of 8:2.

**Figure 3:**
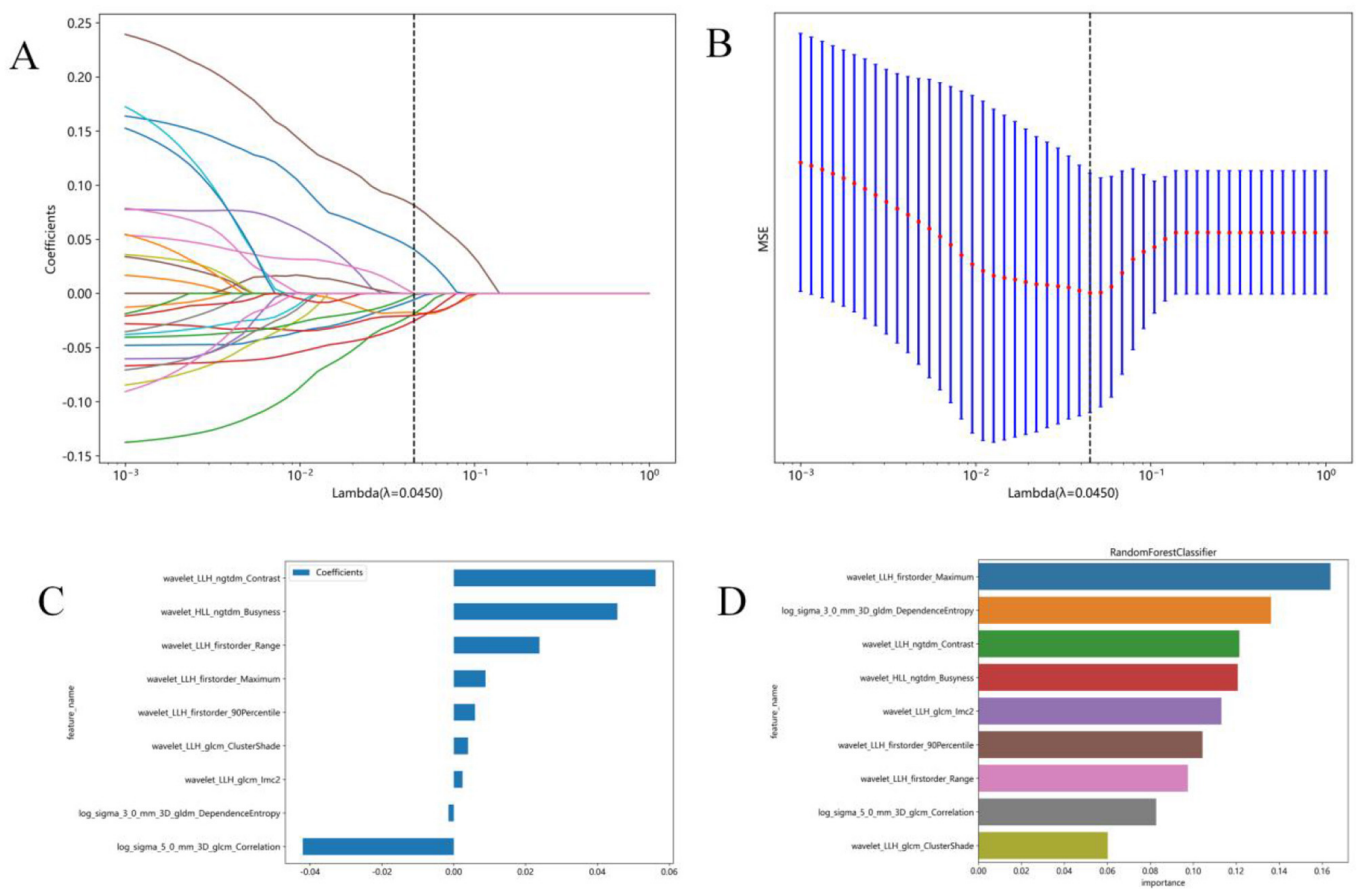
Radiomics feature selection using the least absolute shrinkage and selection operator (LASSO) regression model. **(A)** Tuning parameter (λ) selection in the LASSO model was performed by 10-fold cross-validation using minimum criterion. The optimal λ values are indicated by *dotted vertical lines*. A λ of 0.0045 was chosen. **(B)** LASSO coefficient profiles of 1,197 radiomics features were generated vs. the selected log λ values using 10-fold cross-validation. **(C)** Nine radiomics features with non-zero coefficients were selected. **(D)** Feature weights of the random forest model.

Due to the high complexity of the extracted features, there was a risk of overfitting in the analysis. Thus, employing the Lasso algorithm was necessary to refine the model by constructing a penalty function. This function compresses some coefficients to zero, aiming to enhance the predictive accuracy and interpretability of the model. The least absolute shrinkage and selection operator (LASSO) is a popular high-dimensional data analysis method used to improve prediction accuracy and interpretation. By estimating the regression coefficients for every feature and successively shrinking them, LASSO avoids inflating the estimated coefficients, resulting in superior predictive performance and eliminating irrelevant features. Subsequently, the optimal λ was used to determine the number of features and select the most predictive subset, whose corresponding coefficients were evaluated (Figure 3). The radiomic texture was computed by summing the selected features weighted by their coefficients.

The final selected features were utilized to construct models employing three machine learning algorithms: K-Nearest Neighbors (KNN), Random Forest, and Extra Trees. Additionally, a 10-fold cross-validation process was implemented on the data. Ultimately, the best-performing model was chosen for presentation. All feature selection and classification operations were conducted using Python. Radiomic features were derived from correlation filters, and the most robust, non-redundant, and predictive features were selected using LASSO.

### 2.6 Prediction model building

**Figure 4:**
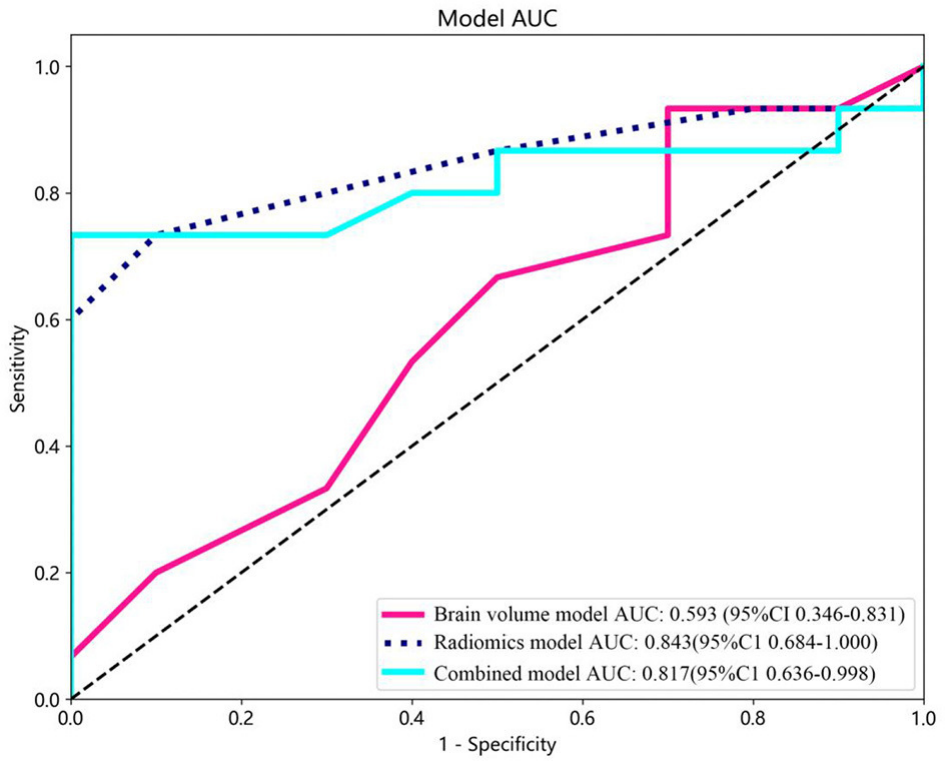
Receiver operating characteristic (ROC) curves of the brain volume, radiomics, and combined models. CI, confidence interval.

Brain volume features were selected using LASSO. Subsequently, prediction models were created incorporating brain volume markers and radiomic texture for final interpretation and analysis. The combined model was developed in conjunction with brain volume markers and radiomic texture. The diagnostic efficacy of the prediction models were assessed in the test set, and receiver operating characteristic (ROC) curves were utilized for evaluating the diagnostic efficacy of three models (Figure 4).

### 2.7 Clinical usefulness and calibration curves

**Figure 5:**
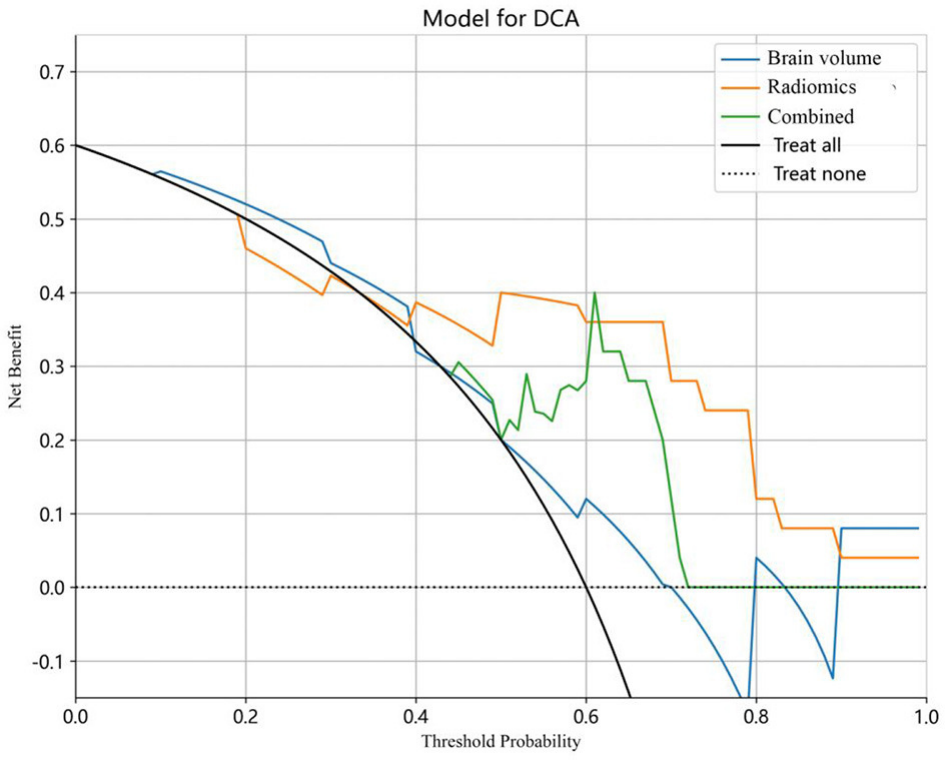
Decision curve analysis (DCA) for the predictive models. The *y*-axis stands for the net benefit, and the x-axis represents the threshold probability. The radiomics model achieved a larger net benefit compared to the brain volume model and the combined model.

The decision curve analysis (DCA) was utilized to validate the clinical relevance of this study, providing insights into the net benefit derived from choosing a specific threshold probability. DCA quantified the clinical value by estimating the net benefits based on threshold probabilities, with net benefit representing the true positives minus the fraction of false positives, weighted by the relative harm of false-positive and false-negative results. The threshold probability, Pt, determined the point where the expected benefit of treatment equaled the expected benefit of avoiding treatment (Figure 5).

**Figure 6:**
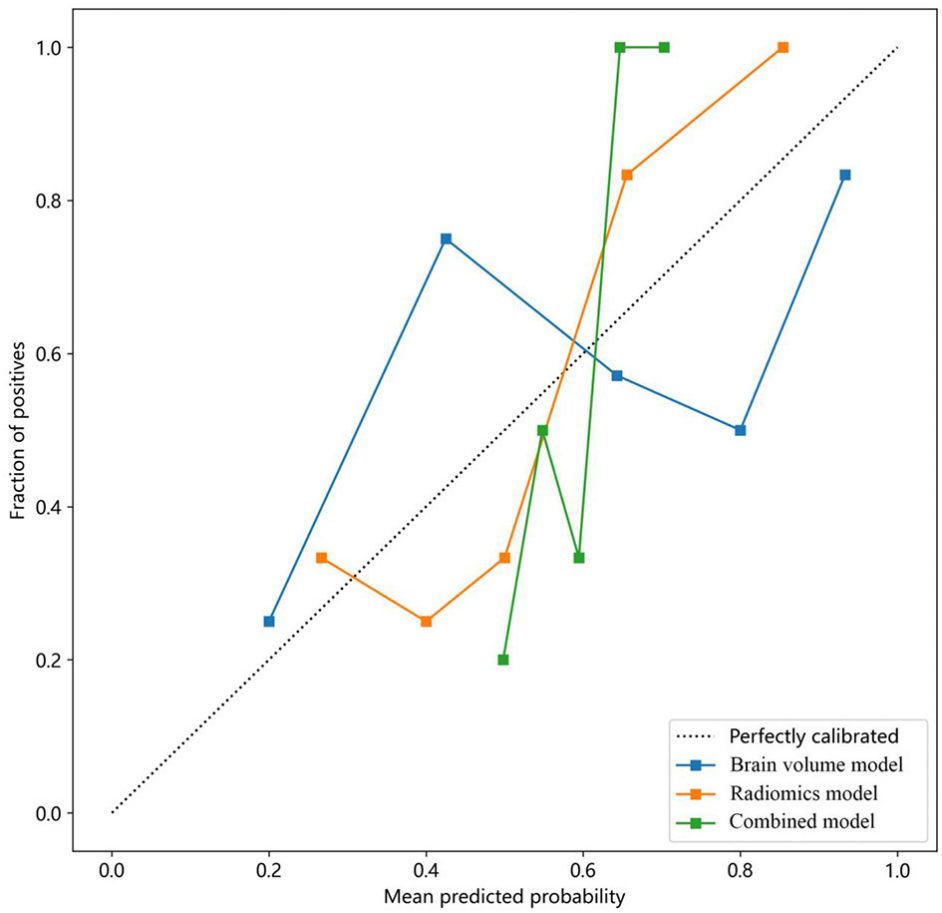
Calibration curves of the brain volume, radiomics, and combined models for predicting CSVD. The *y*-axis represents the actual incidence rate of CSVD. The *x*-axis represents the predicted risk of CSVD. The *diagonal dotted line* represents the prediction of an ideal model. A closer fit to the *diagonal dotted line* indicates a more accurate prediction. CSVD, cerebral small vessel disease.

Moreover, calibration curves were employed to assess the calibration of the radiomics model. Additionally, the Hosmer–Lemeshow test was performed to evaluate the calibration of each model, demonstrating the accuracy of predicted risks of CSVD by comparing them to the observed outcomes of CSVD (Figure 6).

### 2.8 Statistics

In this study, missing value processing was performed as follows: for numeric features, the mean value was utilized for filling. Statistical analyses were conducted using Python software (Version 3.7; https://www.python.org). Normal distribution of variables for the control subject group and CSVD group was tested. We tested normality of variance using the Kolmogorov-Smirnov Test. In a two-tailed analysis, a *p*-value < 0.05 was considered statistically significant. An independent sample *t*-test was employed to evaluate the measurement variables, and the chi-square test and Fisher exact test were used to compare the categorical variables. The predictive performance of the models was assessed using ROC analysis. The DeLong test, a non-parametric approach, was utilized to compare the areas under two correlated ROC curves, determining if there was a statistically significant difference in the predictive accuracy of different diagnostic tests.

## 3 Results

### 3.1 Patient characteristics

The patient characteristics for the CSVD and healthy control groups are presented in Table 1. No significant differences in sex, age, or education were observed (*P* > 0.05). The mean age was 52.57 ± 4.88 years in the control subjects and 52.66 ± 5.15 years in the CSVD group. However, there were statistically significant differences in TIV, GM, WM, WMH, and hippocampal volume, with lower values observed in the CSVD group compared to the control subjects.

**Table 1:** Demographic information in this study.

	**Normal controls (*n =* 99)**	**CSVD (*n =* 145)**	***T*-value/*X*^2^**	***P-*value**
Age (year, mean ± SD)	52.576 ± 4.885	52.662 ± 5.151	−0.131	0.896
Male (*n*, %)	44 (44%)	65 (44.8%)	0.003	0.953^a^
Education (year, mean ± SD)	11.995 ± 2.617	11.754 ± 3.898	0.537	0.592
MOCA (mean ± SD)	27.172 ± 1.069	21.352 ± 4.161	13.606	<0.001
BMI (mean ± SD)	23.26 ± 2.072	24.511 ± 2.81	−3.782	<0.001
Fazekas grade (%)			19.152	<0.001^b^
0	15 (15.2)	2 (1.4)		
I	66 (66.7)	106 (73.1)		
II	15 (15.2)	25 (17.2)		
III	3 (3.0)	12 (8.3)		
LIs (%)	9 (9.1)	17 (11.7)		0.673^a^
CMBs (%)	16 (16.2)	25 (17.2)		0.863^a^
TIV (mean ± SD)	1494.574 ± 130.124	1451.171 ± 128.543	2.577	0.011
GM (mean ± SD)	580.013 ± 42.034	562.161 ± 43.145	3.207	0.002
WM (mean ± SD)	510.743 ± 49.454	493.417 ± 51.715	2.615	0.009
CSF (mean ± SD)	402.597 ± 74.695	393.38 ± 92.148	0.827	0.409
WMH (mean ± SD)	1.221 ± 1.002	2.213 ± 3.781	−2.548	0.011
H-VOL (cm^3^, mean ± SD)	7.416 ± 0.696	7.129 ± 0.663	3.246	0.001

CSVD, cerebral small vessel disease; MOCA, montreal cognitive assessment; BMI, body mass index; TIV, total intracranial volume; GM, gray matter; WM, white matter; CSF, cerebrospinal fluid; WMH, white matter hyperintensities; H-VOL, hippocampus volume; SD, standard deviation.

Statistics were analyzed with independent samples t-test, unless otherwise indicated.

^a^Fisher exact test or chi-square test.

^b^Adjusted chi-square test.

### 3.2 Radiomics feature and models building

In this study, 1,197 features were extracted from 3D MRI T1-weighted images, including 234 first-order features, 286 GLCM features, 182 GLDM features, 208 GLRLM features, 208 GLSZM features, 65 NGTDM features, and 14 shape features. Finally, 9 radiomics features with non-zero coefficients were selected (Figure 3). Three machine learning algorithms were utilized to construct the radiomics model. Among these models, the random forest model achieved the best ROC on the testing set. In the training set, the area under the ROC curve (AUC) was 0.999 [95% Confidence Interval (CI): 0.998–1.000], with an accuracy, sensitivity, and specificity of 98.2%, 96.9%, and 100%, respectively. In the testing set, AUC was 0.843 (95% CI: 0.684–1.000), with an accuracy, sensitivity, and specificity of 80%, 73.3%, and 90%, respectively (Table 2).

**Table 2:** Classification performance.

**Model**	**Accuracy**	**AUC**	**95%CI**	**Sensitivity**	**Specificity**	**PPV**	**NPV**	**Task**
KNN	71.7	0.760	0.699–0.821	80.8	58.4	0.739	0.675	Train
KNN	72.0	0.763	0.581–0.945	66.7	80.0	0.833	0.615	Test
Random forest	98.2	0.999	0.998–1.000	96.9	100	1.000	0.957	Train
Random forest	80.0	0.843	0.684–1.000	73.3	90.0	0.917	0.692	Test
Extra trees	99.5	1.000	0.999–1.000	99.2	100	1.000	0.989	Train
Extra trees	76.0	0.767	0.570–0.963	66.7	90.0	0.909	0.643	Test

KNN, K-Nearest Neighbors; CI, confidence interval; PPV, positive predictive value; NPV, negative predictive value; AUC, area under the receiver operator characteristic curve.

Furthermore, the feature wavelet_LLH_first order_Maximum obtained the highest weight among the selected features using the random forest machine learning algorithm (Figure 3). The results of the Random Forest model and Extra Trees model displayed significant differences, as evidenced by the DeLong test (*P* = 0.047) (Table 3).

**Table 3:** Delong test of KNN, random forest and extra trees model in test set.

	**Random forest vs. KNN model**	**Random forest model vs. extra trees model**	**KNN model vs. extra trees model**
*P-*value	0.083	0.047	0.874

KNN, K-Nearest Neighbors; vs., versus.

### 3.3 Combined model build and correlation analysis

In this study, brain volume features, including WHM, TIV, GM, and hippocampal volume, were selected using LASSO to construct the brain volume model, which was then combined with hippocampal omics textures to form the combined model. The performance of the brain volume model, radiomics model, and combined model in the training and test sets is summarized in Table 4. The AUC of the brain volume model was 0.593 (95% CI: 0.356–0.831), the AUC of the combined model was 0.817 (95% CI: 0.635–0.998), and the AUC of the hippocampal omics model was 0.843 (95% CI: 0.684–1.000) (Table 4, Figure 4). The DeLong test indicated no statistical difference in the AUC values among the three models (Table 5). The brain volume model exhibited higher sensitivity (93.3%) compared to the radiomics model (73.3%), whereas the radiomics model had higher specificity (90.0%) than the brain volume model (30.0%). Additionally, the combination of imaging markers and hippocampal texture did not enhance diagnostic efficacy compared to the individual model (*p* > 0.05) (Table 4).

**Table 4:** The results of different models.

**Model**	**Accuracy (%)**	**AUC**	**95%CI**	**Sensitivity (%)**	**Specificity (%)**	**PPV**	**NPV**	**Task**
Brain volume model	98.2	0.998	0.995–1.000	99.2	96.6	0.977	0.989	Train
Radiomics model	98.2	0.999	0.998–1.000	96.9	100	1.000	0.957	Train
Combined model	99.1	1.000	0.999–1.000	98.5	100	1.000	0.978	Train
Brain volume model	68.0	0.593	0.356–0.831	93.3	30.0	0.667	0.750	Test
Radiomics model	80.0	0.843	0.684–1.000	73.3	90.0	0.917	0.692	Test
Combined model	84.0	0.817	0.635–0.998	73.3	100	1.000	0.714	Test

CI, confidence interval; PPV, positive predictive value; NPV, negative predictive value; AUC, area under the receiver operator characteristic curve.

**Table 5:** Delong test in test set.

	**Brain volume model vs. radiomics model**	**Brain volume model vs. combined model**	**Radiomics model vs. combined model**
*P-*value	0.069	0.062	0.530

### 3.4 Clinical usefulness and calibration curves

The combined model reached a threshold probability of 0.7. The hippocampal omics model demonstrated a greater net benefit at the threshold probability compared to both the brain volume model and the combined model (Figure 5). The calibration curve of the radiomics model depicted the most consistent predicted probabilities among the three models with the observed frequencies, as the curve closely followed the diagonal reference line (Figure 6).

## 4 Discussion

The objective of this study was to develop a prediction model based on hippocampal texture for predicting cognitive impairment in middle-aged patients with CSVD. Nine texture features were screened to construct the prediction model. The findings indicated that the hippocampal omics model exhibited high diagnostic performance in predicting cognitive function impairment in middle-aged CSVD compared to the brain volume-based imaging marker model. Furthermore, the combination of the two models did not significantly enhance the predictive efficacy of the hippocampal omics model.

The study utilized a Random Forest Classifier to develop a radiomics model that achieved 80% accuracy in distinguishing CSVD patients from normal subjects, with a sensitivity of 73.3% and specificity of 90%. This model demonstrates good accuracy and specificity, making it a valuable diagnostic tool. It is well-established that hippocampal atrophy in AD patients primarily results from the accumulation of Tau and amyloid deposits, leading to the formation of characteristic neurofibrillary tangles, which subsequently disrupt the structure and function of hippocampal neurons and nerve fibers (20). The etiology of hippocampal atrophy in CSVD patients is more complex, potentially involving microcirculatory disturbances in addition to neurodegeneration (1). Despite the different pathological and physiological mechanisms of hippocampal atrophy in the two diseases, it has been shown that using hippocampal volume and textural features reflecting microstructural damage is effective for disease prediction (20, 21). Khan et al. achieved 80.7% classification accuracy in differentiating AD from normal controls using hippocampal volume (21). Furthermore, radiomic features can capture more subtle changes in microstructure and microenvironment compared to volume features. Luk et al. illustrated that hippocampal occupancy yielded an AUC of 0.843, with 77.6% sensitivity and 79.6% specificity, while texture achieved an AUC of 0.928, with 88% sensitivity and 84.9% specificity in predicting MCI conversion to AD (15). Feng et al. demonstrated that radiomic features enabled the differentiation of AD from normal controls with an accuracy of 86.75% (specificity = 88.89% and sensitivity = 84.21%) and an AUC of 0.93 (22). Leandrou et al. showed that a radiomics model achieved 84.7% classification accuracy in differentiating AD from normal controls, with a sensitivity of 0.799, specificity of 0.878, and an AUC of 0.91, respectively (23). The accuracy of our model is similar to that of the aforementioned study. However, the sensitivity and specificity exhibit more variability across different studies, indicating potential influence from diverse algorithm models. The radiomics model in this study achieved an AUC of 0.843, signifying strong diagnostic value. Overall, our findings emphasize the potential of hippocampal texture characteristics as imaging biomarkers for predicting cognitive impairment in middle-aged CSVD patients.

The second important finding of this study is that, while there were statistical variances in hippocampal volume, TIV, GM, and WM between the two groups, the imaging marker model exhibited significantly lower efficacy in predicting cognitive impairment in middle-aged CSVD patients compared to the hippocampal textural model. Specifically, the imaging marker model achieved an accuracy of 68%, an AUC of 0.593, a sensitivity of 93.3%, and a specificity of 30%. Prior studies have asserted that brain atrophy and cortical thinning are crucial indicators of aging, neurodegenerative diseases, and CSVD (1, 24). Fein et al. demonstrated that hippocampal and cortical atrophy were the strongest predictors of subcortical ischemic vascular disease (20). Other studies have shown that brain atrophy outperforms all other lesions in predicting cognitive outcomes and disabilities in CSVD (20, 24). In our study, we observed that a brain volume model had lower AUC and specificity but with a high sensitivity of 93.3%. There are several reasons that may explain this result. From the neurodegeneration perspective, brain atrophy occurrence in CSVD patients is closely related to age and interacts with the total load of CSVD, and the lower enrollment age and CSVD load in this study may reduce the incidence of brain atrophy (9, 25). From the vasogenic pathology perspective, the middle-aged CSVD population has a high cerebral perfusion reserve, leading to a relatively small impact of CSVD on cerebral perfusion reduction in middle age, causing subtle secondary structural changes (26, 27). In this study, the lower enrollment age and CSVD burden were linked to fewer brain morphological changes, consequently reducing the predictive efficacy of brain atrophy on cognitive function. Furthermore, the morphological features of brain atrophy have high sensitivity and low specificity to CSVD cognitive function. Therefore, further investigation of the extent of brain atrophy in different age groups of CSVD patients is essential.

WHM and brain atrophy are considered the primary neuroimaging features of cerebral small vessel disease based on the 2023 neuroimaging standards (1). WMH is also among the most prevalent and prominent changes in elderly individuals (28). Previous studies have established that WMH is linked to a significant decline in overall cognitive function (29, 30). Our analysis corroborates the findings of prior research. The severity of WMH correlates with further declines in cognitive scores; Zeng et al. observed that participants with a Fazekas score of 3 or higher exhibited significant impairments in both cognitive functions and in their white matter microstructure (31). Moreover, Wang et al. demonstrated the heterogeneity of WMH concerning its severity and its impact on cognitive impairment (32). However, in this study, the lower WMH load in the enrolled CSVD population diminished the predictive efficacy of WMH.

Previous studies have highlighted the emergence of radiomics as a promising technology capable of enhancing disease diagnosis and prediction by extracting high-throughput quantitative features from medical images of the hippocampus in Alzheimer's disease (33–36). In our present study, we extracted classes of quantitative radiomic features from the hippocampus to assess microenvironmental changes in patients with CSVD. We selected 9 higher texture features, including first-order (*n* = 3), GLDM (*n* = 1), NGTDM (*n* = 2), and GLCM (*n* = 3) among the features. In our study, the feature “wavelet_LLH_first order_Maximum” exhibited the highest weight among the selected features. Wang et al. have demonstrated that GLCM and GLSZM features were associated with the Mini-mental State Examination scores in diagnostic models for AD and aMCI (35, 37). Furthermore, GLCM analysis serves as a highly sensitive method for analyzing Granule Neurons of the Hippocampal Dentate Gyrus following cortical injury (38). We utilized GLCM and GLSZM features to construct our model. In the field of medical image diagnostics, various types of features, including first-order, GLCM, GLDM, and GLRLM, are commonly combined with other feature types, such as log-sigma original and wavelet features (39). The use of wavelet transform enables the investigation of a range of scales, facilitating the enhancement of subtle contrast variations between lesions and normal tissues (40). This approach is preferred over using them individually. Although ongoing research is exploring the correlation between multiple radiomic features and pathology, such studies are still in their preliminary stages, and there are currently insufficient research results to explain the interpretability of these features.

In the clinical setting, the changes in clinical symptoms in middle-aged individuals with CSVD are not significant. Neuropsychological tests necessitate a high degree of patient cooperation, and some patients may struggle to accurately complete the tests. Radiomic features offer a more objective and convenient biomarker for predicting cognitive impairment in CSVD. Specifically, 3D-T1 imaging is commonly used in routine brain examinations, allowing the 3D T1-based hippocampal texture omics model to be readily employed for the primary prevention of cognitive impairment in patients with CSVD.

However, this study has several limitations. Firstly, it was conducted at a single center with a small sample size, potentially constraining the generalizability of the results. Due to the limited sample size, cognitive impairment was not classified into mild, moderate, and severe categories, and there was insufficient neuroimaging data for analysis. In the subsequent phase, we aim to include a larger sample size and more neuroimaging data to establish deep learning models for examining the relationship between the hippocampus and cognitive impairment in CSVD. Additionally, as the study was conducted at a single center, the images were not preprocessed, emphasizing the need for external validation through multi-center studies with larger sample sizes. Secondly, the study involved predominantly Chinese participants, necessitating caution in generalizing the results to other ethnic groups due to potential differences. Thirdly, the developed automatic segmentation algorithm for the hippocampus was not compared with other existing algorithms. Fourthly, while our study achieved favorable results with the Random Forest classifier in terms of AUC and accuracy, selecting the best-performing fold in 10-fold cross-validation may have obscured the model's performance variability across other folds. To mitigate this limitation, we will explore model performance variability more thoroughly using stricter cross-validation strategies and a wider range of evaluation metrics in future work. Fifthly, a clear distinction between CSVD and AD remains challenging due to their mutual high-risk factors and shared disease pathways. However, the potential confounding effect of AD on our study results is mitigated by the fact that our subjects were middle-aged adults with high MoCa scores indicative of minimal AD risk, and we conducted a thorough medical history collection to exclude clinical manifestations of AD at enrollment. Finally, the clinical interpretability of our proposed prediction method is lacking, in our upcoming research efforts, we plan to utilize the feature importance assessment technique to accurately quantify the contribution of each feature to the prediction outcomes.

## 5 Conclusions

In conclusion, our findings indicate that radiomics analysis of hippocampal texture can effectively predict cognitive impairment in middle-aged patients with CSVD. However, the combined model utilizing radiomic signatures, TIV, GM, WMH, and hippocampal volume values does not significantly improve diagnostic efficacy. Furthermore, a radiomics model based on hippocampal structure represents a convenient and reliable protocol for the primary assessment of cognitive impairment in middle-aged CSVD patients.

## Data Availability

The raw data supporting the conclusions of this article will be made available by the authors, without undue reservation.
